# Imaging features of volume replacement using an acellular dermal matrix in oncoplastic breast conserving surgery: A case report

**DOI:** 10.1016/j.radcr.2022.03.003

**Published:** 2022-04-13

**Authors:** Chae Bin Lee, Young-Seon Kim, Seung Eun Lee

**Affiliations:** Department of Radiology, Yeungnam University Hospital, Yeungnam University College of Medicine, Daegu, Korea

**Keywords:** Acellular dermal matrix, Breast, Carcinoma, Imaging, ADM, Acellular dermal matrix, MRI, Magnetic resonance imaging

## Abstract

An acellular dermal matrix (ADM) is a type of allograft that can be made from human, bovine, or porcine dermis and is used to support or reconstruct soft tissue. During breast reconstructive surgeries, ADMs are widely used to partially cover breast implants following a mastectomy to correct for insufficient subcutaneous tissue. Recently, ADMs have been used as a filling material for volume replacement in oncoplastic breast conserving surgery. In this report, we present the case of a female, middle-aged patient who underwent breast conserving surgery with volume replacement using an ADM.

## Introduction

The gold standard for the treatment of small breast cancer includes breast conserving surgery with adjuvant radiation therapy [1]. However, 4%-20% of all patients who undergo breast conserving surgery remain dissatisfied with their cosmetic outcomes [2]. Recently, there has been an increase in the use of oncoplastic surgery in breast cancer treatment, to improve patients’ quality of life. Oncoplastic procedures combine oncologic and plastic surgery techniques to achieve both oncologic safety and better cosmetic outcomes [2]. There are two main techniques in oncoplastic breast conserving surgery: volume displacement and volume replacement. In patients with small- to medium-sized breasts who undergo large-volume resection breast conserving surgeries, the volume replacement technique provides better esthetic results [3]. Several studies have reported that an acellular dermal matrix (ADM) can be used as volume replacement to fill the defect created after a lumpectomy [4], [5], [6]. Herein, we present a case of breast conserving surgery with volume replacement using an ADM.

## Case report

**Fig. 1:**
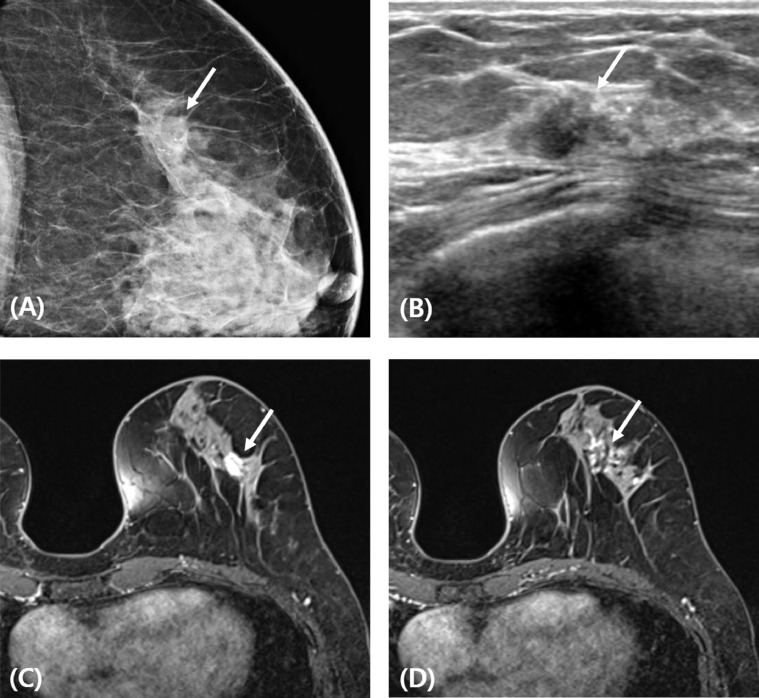
Preoperative imaging findings. (A) An irregularly shaped mass with intra- and extra tumoral microcalcifications (arrow) on mammogram. (B) An irregularly shaped mass and irregular ductal change with intraductal calcifications (arrow) on ultrasound. (C,D) MR images demonstrate an approximately 1.3-cm irregularly shaped mass and focal non-mass enhancement (arrows) with a total extent of approximately 3.5 cm.

**Fig. 2:**
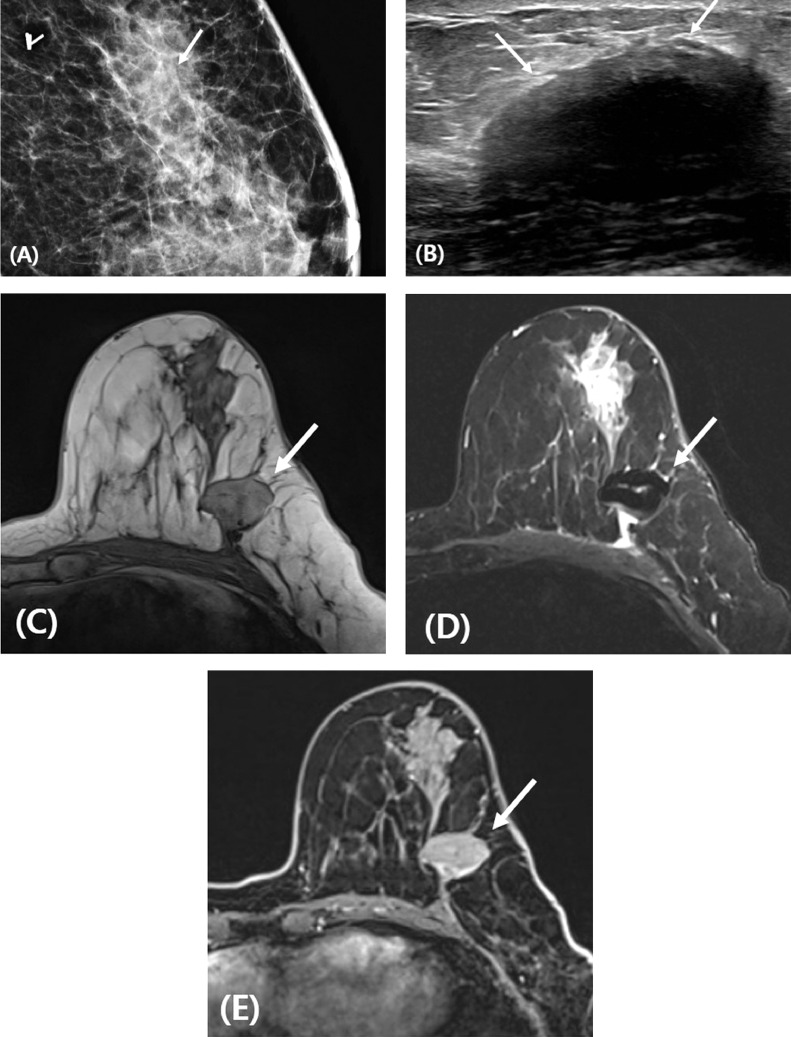
Volume replacement with an acellular dermal matrix (ADM) after right breast-conserving surgery. (A) The ADM is an oval shaped, circumscribed, isodense mass (arrow) on mammogram. (B) The ADM is a hypoechoic mass with posterior acoustic shadowing (arrows) on ultrasound. (C-E) MR images show the ADM (arrows) with iso-signal intensity on the T1-weighted image (C), low signal intensity on the fat-suppressed T2-weighted image (D), and no enhancement on the contrast-enhanced, fat-suppressed, T1-weighted image (E).

A 59-year-old woman was diagnosed with invasive ductal carcinoma of the left breast. Ultrasound revealed an irregularly shaped mass and irregular ductal changes with intraductal calcifications. On preoperative breast magnetic resonance imaging (MRI), irregularly shaped mass and focal non-mass-like enhancement with a total extent of approximately 3.5 cm were found (Fig. 1). The patient underwent breast conserving surgery with sentinel lymph node biopsy. The weight of the removed breast tissue was 25.4 g. The mass was excised with a negative tumor margin and the lumpectomy defect was filled with an ADM (Megaderm®, L&C bio, Seoul, Korea). The ADM was rolled into a cylindrical shape to fill the lumpectomy defect. One-year post-surgery, follow-up mammography and ultrasonography revealed that the inserted ADM had formed a complex without any significant complication. On breast MRI, the inserted ADM appeared as a single mass without contrast enhancement (Fig. 2).

## Discussion

In patients with smaller breast sizes and large lumpectomy defects, volume replacement techniques using autologous tissue flaps can be performed. However, these procedures can result in complications such as a donor site scars or deformities [3]. Partial breast reconstruction using filling materials may be a superior technique to reduce the risk of complications and achieve positive cosmetic results [6].

An ADM is a type of allograft made from human, bovine, or porcine origin that has been processed and sterilized to remove cells and antigenic components and prevent a host immune response [7,8]. ADMs are increasingly used as fillers in breast conserving surgery. Recent studies have reported that volume replacement with ADMs was a safe and cosmetically beneficial procedure [4], [5], [6].

On mammography, an inserted ADM appears as an isodense mass, and may resemble postoperative scars, seromas, or hematomas [8]. On ultrasound, ADMs can appear in various forms that reflect the continuum of vascularization and incorporation of the ADMs into the host. They may appear as iso-to-hypoechoic masses with a folded, sheet-like structure with posterior acoustic shadowing, and with or without color flow [7,8]. On MRI, an ADM shows iso-signal intensity to glandular tissue in a non fat-satured T1-weighted image, and low signal intensity in a fat-saturated T2-weighted image with none to mild dynamic contrast enhancement [4,^8^,9].

ADMs are increasingly used in BCS for volume replacement [10]. In postoperative surveillance, early and accurate diagnosis of recurrence is crucial. The fibro genetic action induced by ADMs and their partial reabsorption may lead to a misdiagnosis during postoperative surveillance [10]. Familiarity with postoperative imaging findings of ADMs used for volume replacement may help to distinguish tumor recurrence and avoid misdiagnosis.

## Patient consent

Written informed consent for publication was obtained from the patient.

## Ethics approval

This study was approved by the Yeungnam University Hospital Institutional Review Board (IRB No. YUMC 2021-10-039) and the requirement for informed consent was waived.
